# Are mental disorders related to disbelief in free will? A systematic review

**DOI:** 10.1186/s13643-021-01621-9

**Published:** 2021-03-16

**Authors:** Maria E. Moreira-de-Oliveira, Gabriela B. de Menezes, Samara dos Santos-Ribeiro, Luana D. Laurito, Ana P. Ribeiro, Adrian Carter, Leonardo F. Fontenelle

**Affiliations:** 1 D’Or Institute for Research and Education, Rio de Janeiro, Brazil; 2 Obsessive, Compulsive, and Anxiety Spectrum Research Program, Institute of Psychiatry, Federal University of Rio de Janeiro, Rio de Janeiro, Brazil; 3 Turner Institute for Brain and Mental Health, Monash University, Clayton, Victoria Australia

**Keywords:** Belief in free will, Psychiatric disorders, FAD, FWDS

## Abstract

**Background:**

The nature and existence of free will have been debated for centuries. Since some psychiatric disorders are known to interfere with one’s ability to control their actions and thoughts (e.g., schizophrenia), the investigation of the psychiatric facet of free will beliefs seems to be relevant. In this systematic review, we were interested in clarifying if and how having a mental disorder affects individuals’ beliefs in free will by comparing psychiatric vs. non-psychiatric samples.

**Methods:**

A systematic search of MEDLINE, Web of Science, EMBASE, and PsycINFO databases was performed between 04 and 09 November 2020. The search strategy included “free will” and related constructs and terms related to DSM-5 mental disorders characterized by psychotic, compulsive, avoidant, or impulsive symptoms. Eligible designs of studies included case-control and cohort studies. Study selection took place in committee meetings consisting of six researchers. Quality assessment of the selected studies was performed through the Joanna Briggs Institute Appraisal Checklist for Case Control Studies.

**Results:**

After removing duplicates, a total of 12,218 titles/abstracts were screened. Inclusion and exclusion criteria were followed, and three articles were eventually selected.

**Conclusions:**

It is not possible to provide unequivocal confirmation that having a mental disorder can or cannot affect someone’s belief in free will. Studies with different mental disorders should be conducted in this field.

**Systematic review registration:**

PROSPERO CRD42018109468.

## Background

Although debates on the existence of free will have occurred over centuries, it is generally understood as the ability to make free choices [1]. However, diverging philosophical views stand out. For instance, libertarians argue that humans have free will by assuming that our decisions have their origins within us, which means that people have the ability to make their own choices and could always act otherwise [2]. In contrast, determinism holds that free will is an illusion, as all human behaviors can be explained as being a consequence of an antecedent event (e.g., a psychological, biological, evolutionary, or environmental circumstance) [3].

Consistent with the deterministic view, seminal experiments show that individuals become aware of their decisions a few milliseconds after their decision occurs [4]. However, if the deterministic perspective holds true, a natural question is whether we can be morally responsible for our actions. The major positions on this issue can be split into those who argue that free will and determinism cannot coexist (incompatibilists) and those who posit that we can be both free and responsible agents (compatibilists), i.e., even in a deterministic condition, we can be hold morally responsible for our actions [5].

Regardless of these different views about the concept of free will, the fact that many people believe in this construct turned the attention of some scholars towards the study of free will beliefs. As researchers developed tools to explore and measure people’s belief in free will [6–11], greater interest in the impact of these beliefs on people’s lives ensued [3, 12–16]. Studies in this field showed that a stronger belief in free will was associated with better self-control [17], better work performance [18], increased helping behavior and less aggression [19], less cheating behavior [20], and less conformity [21]. Thus, regardless of whether free will does or does not exist, it has been suggested that believing in the existence of free will leads to several positive outcomes and a greater sense of agency [22].

Since some psychiatric disorders are known to interfere with one’s agency, studying the psychiatric facet of free will beliefs seems to be relevant. For instance, certain individuals with schizophrenia may feel unable to control their own actions and thoughts, which may be felt to be under the external influence [23, 24]. Similarly, there are other examples where a restriction on the experience of free will (expressed as uncontrollable cognitions and/or behaviors) has been hypothesized to exist, including obsessive-compulsive and related disorders (OCRDs), anxiety disorders, and disorders due to addictive behaviors (DAB) [25–27]. Together, these observations suggest that the perception of free will might be somehow affected in disorders with psychotic, compulsive, avoidant, or impulsive symptoms.

Perhaps one of the greatest examples of the potential impact that research on free will can have on the conceptualization of mental disorders can be seen in addictions. For instance, some scholars have argued over whether addictions are bad personal choices or diseases of the brain or behavior [28]. While the brain disease model sees addictive behaviors as a consequence of neurobiological adaptations that “hijack” decisions about drug use, undermining a person’s free will [29–31], defendants of the choice model suggest that people with addiction always have control over their addiction and engage in their addictive behaviors somewhat willingly [32, 33].

It is also possible that beliefs in free will are associated with specific phenotypes within a specific disorder [34], and discussions in this field range from clinical implications to social and legal aspects involving moral responsibility [35].

For the reasons exposed above, spanning evidence of the experiences of impaired free will in patients showing different psychiatric disorders to links between beliefs in free will and various types of social outcomes (e.g., aggression, cheating, conformity, and self-control), we systematically reviewed the patterns of free will beliefs among people with psychiatric disorders characterized by psychotic, compulsive, avoidant, or impulsive symptoms. Rather than focusing on the sense of agency (the ability a person has to detect whether he or she is the cause of an action and not some other agent [36]), this review focus on beliefs regarding free will (which seems to involve a sense of agency and expecting others to behave in morally responsible fashion [22], as measured by the Free Will and Determinism (FAD)-plus [9] or the Free Will Inventory [8] rating scales). Studying free will beliefs in psychiatric patients might shed some light on certain aspects relevant to clinical practice. Improving the understanding of patient experience can not only lead to a better therapeutic relationship, but also enrich and help to develop psychotherapeutic approaches targeting dysfunctional beliefs.

## Methods

### Objective

The aim of this review was to clarify if and how having a mental disorder affects individuals’ beliefs in free will by comparing psychiatric vs. non-psychiatric samples.

### Protocol and registration

This systematic review protocol was registered at the International Prospective Register of Systematic Reviews (PROSPERO) under the number CRD42018109468 [37].

### Eligibility criteria

Study eligibility criteria were based on the following PECOS format: “participants”—an adult population (18 years aged and above); “exposure”—any psychiatric disorder characterized by psychotic, compulsive, avoidant, or impulsive symptoms listed in the Diagnostic and Statistical Manual of Mental Disorders (DSM-5); “comparator”—a control group (individuals without a psychiatric disorder); “outcome”—the difference in free will belief scores between subjects with psychiatric diagnosis and healthy controls, measured by quantitative tools; and “study design”—case-control and cohort studies, published since 1980.

### Information sources

A systematic search was performed between November 04 and November 09 of the year 2020 in the following databases: Web of Science, Ovid MEDLINE, Ovid EMBASE, and Ovid PsycINFO. The search was performed in rounds using title/abstract/keywords fields—the search strategy for each database is presented in Table 1. Experts (three psychiatrists members of the research team—LFF, GBM, and APR) have chosen specific search terms (exposure of interest) to cover the psychiatric disorders characterized by psychotic, compulsive, avoidant, or impulsive symptoms listed in DSM-5. As we were not interested in studies with mixed diagnosis samples (e.g., patients with “anxiety disorders” rather than “generalized anxiety disorder,” “panic disorder,” “social anxiety disorder”), general terms were not included in this search strategy. In addition to the exposure terms, terms regarding the outcome of interest—beliefs in free will—were used in all searches. To cover the literature as much as possible, we have included “free will” MeSH terms and constructs possibly related to the outcome of interest. No constraint of date, language, or document type was adopted. Whenever necessary, supplementary material of selected studies has been examined for further information. In addition, two independent reviewers (SSR and APR) performed hand searches of the selected studies’ reference lists to supplement the database searching.

**Table 1:** Online search features

Platform	Database	Round^a^	Search terms	Combinations (round numbers)
OVID	MEDLINE, EMBASE, PsycINFO	1	free will OR free choice OR determinis^*^ OR indeterminis^*^ OR compatibilis^*^ OR sense of agency OR personal agency OR personal autonomy OR self determination OR freedom	• 1 AND 2 • 1 AND 3 • 1 AND 4 • 1 AND (5 or 6)
		2	psychotic or psychosis or schizo^*^ or delusional or catatoni^*^ or mood or affective or bipolar or cyclothymic or depress^*^ or dysphoric	
		3	anxiety OR panic OR phobi^*^ OR agoraphobia OR hypochondria^*^ OR illness anxiety disorder OR posttrauma^*^ OR acute stress disorder	
		4	obsessive-compulsive OR body dysmorphic disorder OR dysmorphophobia OR hoard^*^ OR trichotillomania OR hair-pulling OR skin-picking OR excoriation disorder OR stereotyp^*^ OR Tourette OR tic^*^ OR binge eating OR avoidant food intake disorder OR restrictive food intake disorder OR anorexi^*^ OR bulimi^*^	
		5	“substance use” OR “drug use”	
		6	addicti^*^ OR substance abuse OR drug abuse OR alcohol OR cannabis OR phencyclidine OR hallucinogen OR inhalant OR opioid OR stimulant OR tobacco OR gambling OR cocaine OR anxiolytic OR sedative OR hypnotic OR impulse-control OR impulsivity OR kleptomania OR pyromania OR attention-deficit	
–	Web of Science	1	“free will” OR “free choice” OR determinis^*^ OR indeterminis^*^ OR compatibilis^*^ OR “sense of agency” OR “personal agency” OR “personal autonomy” OR “self determination” OR freedom	• 1 AND 2 • 1 AND 3 • 1 AND 4 • 1 AND 5
		2	psychotic or psychosis or schizo^*^ or delusional or catatoni^*^ or mood or affective or bipolar or cyclothymic or depress^*^ or dysphoric	
		3	anxiety OR panic OR phobi^*^ OR agoraphobia OR hypochondria^*^ OR “illness anxiety disorder” OR posttrauma^*^ OR “acute stress disorder”	
		4	obsessive-compulsive OR “body dysmorphic disorder” OR dysmorphophobia OR hoard^*^ OR trichotillomania OR hair-pulling OR skin-picking OR “excoriation disorder” OR stereotyp^*^ OR Tourette OR tic^*^ OR “binge eating” OR “avoidant food intake disorder” OR “restrictive food intake disorder” OR anorexi^*^ OR bulimi^*^	
		5	addicti^*^ OR “substance use” OR “substance abuse” OR “drug use” OR “drug abuse” OR alcohol OR cannabis OR phencyclidine OR hallucinogen OR inhalant OR opioid OR stimulant OR tobacco OR gambling OR cocaine OR anxiolytic OR sedative OR hypnotic OR impulse-control OR impulsivity OR kleptomania OR pyromania OR attention-deficit	

^a^For all searches, the title, abstract, and keywords search field was adopted

### Selection of studies

Duplicate titles across databases and studies published before 1980 were removed. Three independent pairs of independent reviewers (LDL and GBM; MEM and LFF; SSR and APR) assessed the articles—those that clearly did not meet the inclusion criteria were excluded. Any disagreement was resolved by discussions within the broad team during weekly meetings that took place between November 2020 and December 2020. Three psychiatrists (LFF, GBM, and APR), two biomedical scientists (MEM and SSR), and one psychologist (LDL) formed the research team.

### Risk of bias in individual studies

The risk of bias in selected studies was evaluated using the Joanna Briggs Institute (JBI) Critical Appraisal Checklist for Case Control Studies [38]. Four reviewers (MEM, SSR, LDL, and APR) independently classified each aspect listed in the checklist as “yes,” “no,” “unclear,” or “not applied” for each selected study. A consensus was reached during our weekly meetings with researchers MEM, SSR, LDL, APR, and LFF in case of doubts or disagreements. If doubts persisted, corresponding authors were contacted for clarification.

### Data collection process

Two independent reviewers (MEM and LDL) collected the necessary information from the selected articles using an extraction template. Any disagreement was resolved during our weekly meetings with researchers MEM, SSR, LDL, APR, and GBM. The following data were planned to be extracted: first author, country of study, demographics (age and gender of both exposed and control participants), recruitment setting, exposure (the mental disorder diagnosis and how it was determined), matching data (variables used to match groups), study design, primary outcome data (mean values of the free will belief scales domains), and secondary outcome data (mean values of disorder severity scales). Related disorders were planned to be subgrouped in the narrative synthesis to solve a potential heterogeneity across studies.

### Data analysis

A narrative synthesis of the study was conducted. A table and a narrative summary were used to present the study settings and its main findings.

## Results

**Fig. 1:**
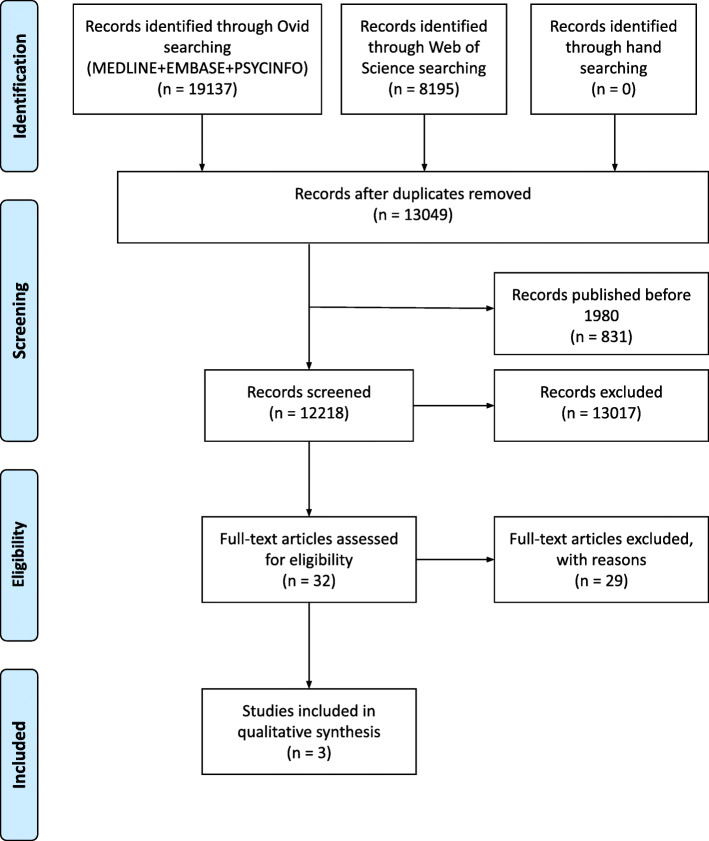
PRISMA flow diagram

The search in all databases yielded 27,332 articles. Duplicate references were removed, leaving 13,049 references. Studies published before 1980 were also removed, and the remaining 12,218 articles had their titles and abstracts screened. Most of the studies were excluded, leaving 32 articles to be further evaluated. Among them, two were not found, and attempts to contact the authors were unsuccessful. Of the remaining 30, only three fitted the eligibility criteria stated in this review. These research steps are shown in the PRISMA diagram (Fig. 1). The three selected articles are listed in Table 2, which also shows the mental disorder diagnosis studied and how it was determined, the recruitment setting, the results of free will belief assessments for both exposed and control participants, the tool used to measure the beliefs, and the design of each study. Although the extraction of matching variables (i.e., variables used to match groups) and secondary outcomes (means on disorder severity scales) was planned, this information was not reported in none of the three studies. In addition, for almost all articles, demographics (age and gender) of both exposed and control participants were not available.

**Table 2:** List of selected studies

	Ent, 2014 ^[1]^		Vonasch, 2017 ^[2]^		van der Salm, 2017 ^[3]^	
Country	USA		USA		NL	
Exposures (*N*)	PD (23), epilepsy (16)		Addiction—alcohol, tobacco, other drugs and activities (not available)		Tics (17), FMD (28), myoclonus (15)	
Exposure determination	Formally diagnosed (self-report)		Yes/no questions about personal history of addiction		Standardized clinical assessment ^[6]^	
Exposure recruitment settings	Online (Mechanical Turk)		Online (Mechanical Turk)		Clinical setting	
Control determination (*N*)	None (35)		Yes/no questions about personal history of addiction (not available)		Unknown (22)	
Control recruitment settings	Online (Mechanical Turk)		Online (Mechanical Turk)		Unknown	
Sociodemographic characteristics *[age (mean, SD); gender (frequency—N of female)]*	PD *[age: not available; gender: 17]* Epilepsy *[age: not available; gender: 6]*	Control *[age: not available; gender: 21]*	Unknown		Unknown	
	Average age of all participants, 32.71					
Outcome assessment tool	FWDS ^[4]^ (G: range 14–70; P: range 8–40)		FWDS ^[4]^ (G: range 14–70; P: range 8–40)		FAD ^[5]^ (free will subscale range 7–35)	
Outcome results (mean, SD)	PD *[G: 55.91 (8.50); P: not available]* Epilepsy *[G: 52.63 (11.72); P: 29.50 (6.71)]*	Control *[G: 59.97 (6.69); P: 32.57 (4.24)*]	ALC *[G: 42.64 (1.31)*^*a*^*; P: 25.05 (0.63)*^*a*^*]* TOB *[not available*^*b*^*]* ODA *[G: 43.51 (1.42)*^*a*^*; P: 24.97 (0.67)*^*a*^*]*	ALC control *[G: 47.43 (0.73)*^a^*; P: 26.76 (0.34)*^*a*^*]* TOB control *[not available*^*b*^*]* ODA control *[G: 47.02 (0.73)*^*a*^*; P: 26.72 (0.34)*^*a*^*]*	Tics *[26.4 (4.1)]* FMD *[26.3 (2.6)]* Myoclonus *[24.9 (3.6)]*	Control *[25.4 (3.8)]*
Type of study	Cross-sectional		Cross-sectional		Cross-sectional	

^a^Standard error

^b^Data not shown by the authors since there were no significant differences in their analysis

^[1]^Ent and Baumeister 2014 [39]

^[2]^Vonasch, Clark, Lau, Vohs, and Baumeister 2017 [40]

^[3]^van der Salm et al. 2017 [41]

^[4]^Rakos, Laurene, Skala, and Slane 2008 [10]

^[5]^Paulhus and Margesson 1994 [42]

^[6]^van der Salm et al. 2013 [43]

One of the three selected articles was entitled *Embodied free will beliefs: Some effects of physical states on metaphysical opinions* authored by Ent and Baumeister (2014). This study compared free will beliefs among people with panic disorder, epilepsy, and healthy controls by using an online version of the Free Will and Determinism Scale (FWDS) [10], which has separate subscales for measuring (i) beliefs in free will in general and (ii) the specific sense of oneself as having free will [39]. Even though beliefs about one’s own free will were marginally diminished (*p* = 0.53) among participants with epilepsy when compared to healthy controls, no significant differences were seen between participants with panic disorder and the healthy control group. People’s beliefs about free will in general, on the other hand, seem to be diminished in both epilepsy and panic disorder conditions when compared to healthy controls [39].

The second one—*Ordinary people associate addiction with loss of free will*—aimed to assess the relationship between people’s belief in free will and their history of addiction [40] by using an online version of the FWDS [10]. Vonasch and colleagues (2017) described that participants who had been addicted to alcohol believe less in free will (both general and personal beliefs) when compared to people who had never been addicted to it. The same pattern of results was observed in participants addicted to other drugs, except for smoking. The authors found, though, that people who tried but failed to quit smoking believe less in their own personal free will.

The third study was entitled *Clinician and patient perceptions of free will in movement disorders: mind the gap*. In this research, van der Salm and colleagues (2017) compared free will beliefs displayed by 60 individuals with hyperkinetic neuropsychiatric disorders [including 28 with functional movement disorders (conversive disorders), 17 with tic disorders, and 15 with myoclonus] to 22 healthy control subjects through the Free Will And Determinism Scale (FAD) [42]. In their analyses, the authors found no significant differences between free will beliefs exhibited by participants in different diagnostic groups and controls.

All the three selected articles were assessed for quality and risk of bias according to the JBI Critical Appraisal Checklist for Case Control Studies [38]. For the final evaluation of these studies by JBI checklist, we also sought other sources for further information (supplementary material, articles listed in reference, and author contact). Table 3 presents the assessment of these articles using the JBI checklist.

**Table 3:** Assessment of primarily selected studies according to the JBI Critical Appraisal Checklist for Case Control Studies

Checklist item	Study		
	[1]	[2]	[3]
1. Were the groups comparable other than the presence of disease in cases or the absence of disease in controls?			
2. Were cases and controls matched appropriately?			
3. Were the same criteria used for the identification of cases and controls?	•	•	•
4. Was exposure measured in a standard, valid, and reliable way?			•
5. Was exposure measured in the same way for cases and controls?	•	•	
6. Were confounding factors identified?		•	
7. Were strategies to deal with confounding factors stated?		•	
8. Were outcomes assessed in a standard, valid, and reliable way for cases and controls?	•	•	•
9. Was the exposure period of interest long enough to be meaningful?			
10. Was appropriate statistical analysis used?	•		•

• = item was assigned as “yes”; [1] = Ent and Baumeister 2014 [39]; [2] = Vonasch, Clark, Lau, Vohs, and Baumeister 2017 [40]; [3] = van der Salm et al. 2017 [41]

## Discussion

This review revealed a small number of studies measuring free will beliefs in individuals with mental disorders. More specifically, only three articles (Table 2) that included a valid assessment of beliefs in free will endorsed by individuals with mental disorders compared the strength or patterns of these beliefs to those shown by participants without mental disorders. Further, at this point, we were unable to exclude the possibility of bias in these articles as per the JBI Critical Appraisal Checklist for Case Control Studies [38] for the lack of sufficient information, including, for instance, whether cases and controls were defined and matched appropriately. None of these studies has investigated the severity of symptoms presented by participants with a mental disorder and how they correlated with their beliefs in free will.

One of the appraised studies found that people’s beliefs in general free will are diminished among those with panic disorder when compared to controls, but no significant differences were seen regarding the beliefs about personal free will between these two groups [39]. However, this study did not describe the methods for matching participants, and it did not confirm participants’ diagnostic status, which relied simply on self-reports of a previous diagnosis. Accordingly, the researchers acknowledged the possibility that some control participants had undiagnosed panic disorder or epilepsy. Although this study suggests that individuals with panic disorder (a proxy of avoidant behaviors) tend to have a weaker belief in general free will than controls; this finding requires more investigation.

Another study described that people who had been addicted to alcohol or a substance/behavior other than alcohol and tobacco believe less in both general and personal free will when compared to people who had never been addicted to these substances. On the other hand, no differences were seen between people who had been addicted to tobacco and people who had not [40]. It seems that, except for tobacco, people with addiction have a decrease in the strength of their beliefs in free will. However, this study did not include a clinical assessment to confirm the addiction diagnosis among participants and/or any way to support the validity of the reported online questionnaire. Thus, it is difficult to guarantee that cases and controls had a well-established boundary between them, i.e., that participants reporting a history of addiction had a diagnosable condition and that healthy controls did not have a substance use disorder. Plus, it is not clear whether the groups were matched properly. Therefore, the relationship between addictions and free will beliefs cannot be completely elucidated based only on these findings.

The third study found that participants with tics had no differences regarding free will beliefs when compared to controls [41], though it is not clear if and how these groups were matched. Studies on tic disorders were included for being listed within the chapter of OCRDs in ICD-11 [44]. However, evidence based on a single study should probably not be considered sufficient to discard the possibility that other mental disorders presenting compulsive behaviors (such as OCRDs and eating disorders) cause or are affected by specific patterns of beliefs in free will.

Although previous narrative reviews suggested that one’s perception of free will is impaired in several psychiatric disorders [29], our review highlights the lack of well-designed studies comparing free will beliefs by individuals with vs. without mental disorders. However, the present report also has several limitations. For instance, one might argue that our search strategy was not ideal for not including all disorders listed in the DSM-5, for excluding studies without a controlled design, and for not including studies published before 1980. Nevertheless, a closer examination of the reasons for our selection criteria supports our choices, as seen below.

Firstly, the current version of the DSM has more than 300 diagnoses, which made the inclusion of all DSM-5 mental disorders into the search strategy challenging. Therefore, we decided to focus on disorders that could be more intrinsically related to distinctive beliefs in free will, such as those presenting psychotic, compulsive, avoidant, or impulsive symptoms. Secondly, our strategy, which excluded studies based on their methodological design, may be considered too strict for a research field that is still growing. However, we thought the latter strategy would allow us to best compare both perspectives (psychiatric and non-psychiatric samples’ beliefs) to identify whether having a mental disorder affects individuals’ beliefs in free will.

Our strategy also allowed the identification of a significant gap in the literature, particularly in relation to psychotic disorders. Although schizophrenia has been traditionally associated with “passivity experiences” (feelings, impulses, or acts imposed by an external agent) [45], no controlled study on beliefs of free will among individuals with schizophrenia was found. The work by Weisman de Mamani et al. [24] represented only an initial attempt in this regard. In the future, studies should clarify whether belief systems in free will differ between psychotic and non-psychotic (or predominantly behavioral) disorders.

Finally, studies published before 1980 were excluded so we could use a homogenous concept of “disorders” regardless of the specific nature of each condition addressed in our study. More specifically, the third version of DSM (DSM-III), published in 1980, firstly introduced the concept of “mental disorder” as a clinically significant behavioral or psychological syndrome that was typically associated with either distress (such as a painful symptom) or disability (impairment in one or more areas of functioning). It was assumed that all “mental disorders” had an underlying behavioral, psychological, or biological dysfunction that was not solely in the relationship between the individual and the society [46].

The fact that believers in free will tend to adopt a coping style that allows them to reframe suffering in a satisfactory manner suggests that the free will concept seems to be relevant for several mental disorders [24]. Thus, rather than simply comparing free will beliefs between different groups, future studies should also investigate whether such beliefs are dependent upon the severity of symptoms, do predict clinical outcomes, or are remediated by therapeutic interventions.

## Data Availability

Not applicable.

## Abbreviations

ALC: Alcohol
DAB: Disorders due to addictive behaviors
DSM-5: Diagnostic and Statistical Manual of Mental Disorders–Fifth Edition
FAD: Free Will and Determinism Scale
FMD: Functional Movements Disorders
FWDS: Free Will and Determinism Scale
JBI: Joanna Briggs Institute
NL: Netherlands
OCRDs: Obsessive-compulsive and related disorders
ODA: Other drugs and activities
PD: Panic disorder
PECOS: Participants, exposure, comparator, outcome, study design
PRISMA: Preferred Reporting Items for Systematic Reviews and Meta-Analyses
PROSPERO: International Prospective Register of Systematic Reviews
SD: Standard deviation
TOB: Tobacco
USA: United States of America
